# Novel behavioral model in evaluating initiation and sustenance of teeth brushing behavior among students pursuing health sciences: a cross-sectional study

**DOI:** 10.12688/f1000research.103077.1

**Published:** 2022-04-04

**Authors:** Dhiraj Panjwani, Mithun Pai, Shweta Yellapurkar, Aayush Anand Poddar, Gururagavendra Rajesh

**Affiliations:** 1 Public health Dentistry, Manipal College of Dental sciences, Mangalore, Manipal Academy of Higher Education,Manipal., Karnataka, 575001, India; 2 Oral Pathology and Microbiology, Manipal College of Dental Sciences, Mangalore. Manipal Academy of Higher Education. Manipal, Mangalore, Karnataka, 575001, India; 3 Public Health, School Of Public health, Boston University, Boston, Massachusetts, 02118, USA

**Keywords:** Behavioral Science, Health behavior, Multi theory model, Oral hygiene, Tooth brushing

## Abstract

**Background: **Oral hygiene maintenance is a crucial and integral feature in determining the overall wellbeing of a person. It has been established that interventions for health promotion at the public health level derived from theoretical models based on social and behavioural sciences have a superior effectiveness as compared to the ones without a theoretical background. Hence a novel behavioral model known as the multi-theory model (MTM) was used to understand two important aspects of health behavior change: (i) Initiation and (ii) Sustenance in twice daily teeth brushing in a university setting with objectives to identify factors effecting MTM in initiation and sustenance of twice daily brushing behavior among students pursuing health sciences and correlating the MTM theory with socio-demographic and behavioral patterns.

**Methods**: The study is an analytical cross-sectional study. Students pursuing Medicine and Dentistry in a University setting were included. A validated questionnaire was designed for this study. Questions were framed to evaluate the constructs of initiation and sustenance of MTM, personality, sleeping habits and demographic corelates of participants. Multiple means between the groups were compared using analysis of variance and a post hoc test. Correlation was established between different domains, the items were then entered for hierarchical multiple regression.

**Results**: Of the 235 participants in the study, 229 completed the questionnaire. There was a significant association between brushing quartiles, professional streams (p<0.001) and academic performance (p<0.001). The hierarchical multiple regression revealed that at stage one, behavioral confidence contributed significantly to the regression model (F (1,227) = 33.227, p<0.001) and accounted for 12.4% of the variation in twice daily brushing.

**Conclusion:** MTM is a good tool in predicting the initiation and sustenance of twice daily brushing behavior among young adults and can form a useful tool in assessing the patterns of brushing behavior in a population.

## Introduction

Dental plaque has been attributed as the leading cause for dental caries and periodontal disease. Loe in 1965 was the forerunner in identifying dental plaque as the cause for development of gingivitis and periodontitis if oral hygiene habits were stopped ^1^ Oral hygiene maintenance is a crucial and integral feature in determining the overall wellbeing of a person. An effective and efficient plaque control program at the individual and group level is the need of the hour in preventive dentistry. ^2^ Tooth brushing is the most important aspect of a competent plaque control program. ^3^ Hence, dental professionals worldwide recommend brushing twice daily as an effective measure for maintenance of oral hygiene. ^2^

There is a progressive focus in research on behavioral theories and their effective and applicable action on initiation and sustenance of a beneficial health behavior among the populace. Sharma *et al*. in the year 2015, designed a novel theory, called the multi-theory model (MTM), which discusses two important aspects of health behavior change the first being initiation and second sustenance. ^4^ The theory imbibes distinct characters such as cognitive, conative and environmental factors from theories that are presently in practice, at group, community and individual levels. ^4^

In this theory, Sharma *et al.* suggest that three constructs govern the initiation of a behaviour. The first construct, originated from the Freire’s model of adult education ^5^ and is called “participatory dialogue”. The second construct is obtained from Bandura’s self-efficacy ^6^ and Ajzen’s perceived behavioral control ^7^ and is termed ‘behavioral confidence’. The last construct regulating health behavior initiation is called “changes in physical environment”. The foundations of this final construct are Bandura’s construct of environment, ^8^ Prochaska’s construct of environmental re-evaluation ^9^ and environmental factors in Fishbein’s integrative model. ^10^ In continuum, there are three separate constructs that regulate sustenance. “Emotional transformation” is the first construct, taking origin from the self-motivation construct of emotional intelligence theory. This domain states that collecting or gathering one’s emotions and translating or reconstructing them towards the orientation of the change in behavior, is imperative to accomplishing the said modification. ^4^^,^^11^^,^^12^ Freire’s adult education model’s praxis contributes to the second construct of health behaviour change continuation, that is termed “practise for change”. ^13^ The third construct of sustenance is “change is social environment”, this is a derivative of construct of environment, facilitating relationships, communal support and so on. ^4^

It has been established time and again, that interventions for health promotion at public health level derived from theoretical models based on social and behavioural sciences have a superior effectiveness as compared to the ones without a theoretical background. ^14^ Hence the objective of this study was to evaluate the factors effecting MTM in initiation and sustenance of twice daily brushing behavior among students pursuing health sciences using a questionnaire derived from the MTM theory and correlating the MTM theory with socio-demographic and behavioral patterns among the participants. This is the first novel attempt to assess the efficacy of the MTM in the field of dentistry.

## Methods

The study was carried out in the city of Mangaluru, Karnataka, India. The study was conducted in June 2019. Participants included undergraduate students from year one to year five pursuing Medicine and Dentistry in a university setting. A cross-sectional design was used to evaluate the attitude of the participants’ towards their oral health and intentions to maintain the habit of brushing two times a day using MTM theory and correlating MTM theory with socio-demographic and behavioral pattern among the participants.

### Ethics

Ethical approval was obtained prior to the study process from Institutional Ethics Committee. (Protocol ref no. 17133).

Sample size was calculated using G*power 3.1 (G*Power, ***RRID***:SCR_013726) (Heinrich-Heine-Universität Düsseldorf, Düsseldorf, Germany) The sample size was estimated using to detect an effect size (d = 0.30) based on 80% power, using two-tailed significance tests and an α = 0.05 which was calculated from pilot study among 30 participants. The final sample size was determined to be 222.

### Instrumentation

A 46-item questionnaire ^32^ was designed using pertinent literature on oral health behaviour. The questionnaire was designed to evaluate MTM theory and was modelled on the questionnaires from Sharma *et al*. and Nahar *et al*. ^11^^,^^15^^,^^16^ The instrument included 19 questions on socio demographic details and oral hygiene habits of the participants. The next 27 questions evaluated the constructs of initiation and sustenance of the MTM and were measured on a five-point Likert scale (1=not at all sure to 4=totally sure). Questions were included in the questionnaire from the ‘Ten Item Personality Inventory’ ^17^ and ‘questions on Sleeping habits (self-constructed and validated (20))’ after suggestions from subject experts after evaluating the instrument during the pilot study.

The questionnaire was created using Google forms, where the questionnaire was shared to the students via a link through a group message on WhatsApp messenger (Meta, Massachusetts, US). The participants filled an informed consent form prior to answering the questionnaire and only those who consented, were included in the study. Those suffering from any medical conditions that restricted them from being able to brush twice daily were excluded from the study.

### Data collection and statistical analysis

The independent t tests and one-way analysis of variance (ANOVA) were used according to the division of groups to compare data between demographic variables, twice daily brushing and MTM scores. Pearson product-moment correlation was used to correlate brushing habits, MTM questionnaire scores with Ten Item Personality Inventory and the questionnaire on sleeping habits. A correlation was established between different domains of MTM, Ten Item Personality Inventory, twice daily brushing and demographic criteria. The items which showed statistical significance were then entered into the block for hierarchical multiple regression. A hierarchical linear regression is a form of a multiple linear regression analysis in which multiple variables are added to the model in separate steps. This was done to statistically control variables (Cofounders) (academic class, academic performance, professional fields), to see whether adding variables significantly improves a model’s ability to predict the outcome (MTM Model) variable to investigate the model. A stepwise regression was then performed ( Table 4), among the significant covariates and the independent variables for these models. All statistical analyses of data were completed using IBM SPSS statistical software version 21.0 (IBM Corp, New York 2012) (RRID: SCR_ 019096) with a significance level p= 0.05.

## Results

Of the 235 participants in the study, 229 completed the questionnaire with 6 questionnaires deemed incomplete for analysis. Participant’s mean age was 20.70 years with standard deviation of 1.6 years. Higher participation was seen from the dental students (70%) as compared to the medical students. The highest responses were received from first (n= 65) and third (n=59) year students followed by second years (n=43), fourth years (n=39) and interns (n=23). The majority of the participants were hostel residents. A small number of participants worked for financial gain in the form of internships and part-time jobs. Table 1 demonstrates there was a significant association between the participants from medical and dental stream (p<0.001). The academic year also showed significant association between tooth brushing and MTM scores, the post hoc test showed that interns showed the maximum association between MTM scores followed by third years and first years. (mean MTM scores according to academic year: First year 82.58, second year 76.98, third year 83.17, fourth year 81.03 and interns 91.09) ^31^

**Table 1.** The demographic data and its association to multi theory model (MTM) scores, Independent t test and one way analysis of variance is used according to the division of groups.

			n	Mean	SD	min	25	75	max	T value	p
MTM score	Course	Medical	68	71.84	17.37	27	60	84	110	-6.557	**<0.001**
		Dental	161	86.68	14.87	27	79	97	115		
MTM score	Residence	Hostel	185	81.72	16.73	27	73	93	114	-0.996	0.320
		Residence	44	84.57	18.26	27	71	98	115		
MTM score	Work	Yes	34	79.71	20.07	27	71	95	108	-727	0.253
		NO	195	82.72	16.5	27	72	94	115		
										**F value**	
MTM score	Academic	<50%	7	71.43	28.21	27	47	93	108		
		50-60%	17	78.59	19.32	49	62	93	110	1.049	0.383
		61-70%	73	82.84	16.23	27	71	95	115		
		71-80%	100	83.41	16.67	27	77	95	110		
		>80%	32	81.75	15.73	53	67.5	91	114		
Times brushing		I year	65	3.28	3.029						
		II year	43	2.65	2.645					1.851	**0.12**
		III year	59	3.95	3.126						
		IV year	39	2.72	2.752						
		Interns	23	3.87	2.943						
MTM score	Year	I year	65	82.58	19.22	27	74	95	115		
		II year	43	76.98	18.18	27	67	88	109		
		III year	59	83.17	15.3	27	74	92	110	2.762	**0.029**
		IV year	39	81.03	16.28	47	69	92	114		
		Interns	23	91.09	9.13	75	84	99	108		

The data were grouped according to the number of times they brushed twice daily in the week into quartiles with participants who did not brush twice daily even once that entire week, participants who brushed twice daily at least one to three times that week, participants who brushed twice daily at least four to six times in that week and participants who brushed twice daily every day of that week were categorized into four groups. Table 2 showed a significant association between brushing quartiles, professional streams (p<0.001) and academic performance. People with higher academic grades were brushing twice daily for more days in a week than with students with lower academic grades. brushing twice daily every day of the week with brushing (p<0.035) no significant association was observed with year of study.

**Table 2.** The demographic data and its association to brushing quartiles, independent t test and one way analysis of variance is used according to the division of groups.

		Count	Mean	Standard Deviation	Percentile 25	Percentile 75	t value	p value
Brushing quartiles	Medical	68	1.9	1.24	1	3	-3.363	**0.001**
	Dental	161	2.52	1.28	1	4		
Brushing quartiles	Hostel	185	2.3	1.29	1	4	-0.802	0.792
	Residence	44	2.48	1.32	1	4		
Brushing quartiles	Working	34	2.18	1.38	1	4		
	NON working	195	2.36	1.28	1	4	-0.744	0.325
							**F value**	
Brushing quartiles	<50%	7	1.86	1.46	1	4		
	50-60%	17	2.06	1.2	1	3		
	61-70%	73	2.15	1.29	1	3	2.635	**0.035**
	71-80%	100	2.48	1.29	1	4		
	>80%	32	2.56	1.32	1	4		
Brushing quartiles	I year	65	2.32	1.32	1	4		
	II year	43	2.02	1.16	1	3		
	III year	59	2.64	1.34	1	4	2.147	0.076
	IV year	39	2.08	1.24	1	3		
	Interns	23	2.61	1.31	1	4		

A one-way ANOVA was carried out to compare the effect of the (independent variable) number of times a participant brushes twice daily during the week on the (dependent variable) MTM scale. As seen in Table 3 there was a significant correlation between the number of times a participant brushes twice daily during the week and the MTM scale (p<0.05) for the three conditions [F (3, 225)=15.699, p<0.001]

**Table 3.** One-way analysis of variance was carried out to compare the effect of the (independent variable) number of times a participant brushes twice daily during the week on the (dependent variable) multi theory model scale.

A: Dunnetts Post hoc test was done as variance was assumed equal(Levene test p=0.264).						
Brushing quartiles		Sum of Squares	df	Mean Square	F	Sig.
First quartile Second quartile Third quartile Fourth quartile	Between Groups	11423.903	3	3807.968	15.669	0.000
	Within Groups	54681.311	225	243.028		
	Total	66105.214	228			

B: Dunnett t (2-sided).						
Brushing quartiles	Brushing quartiles	Mean Difference	Std. Error	Sig.	95% Confidence Interval	
					Lower Bound	Upper Bound
First quartile	Fourth quartile	-16.479 ^*^	2.455	.000	-22.3259	-10.6321
Second quartile	Fourth quartile	-13.603 ^*^	3.538	.000	-22.0316	-5.1761
Third quartile	Fourth quartile	-8.298 ^*^	3.205	.029	-15.9314	-0.6652

^*^ The mean difference is significant at the 0.05 level.

Dunnett’s post hoc test was done as variance was assumed equal (Levene’s test p=0.264). Analysis revealed that participants who brushed twice daily every day in the week reported highly significant values when answering the MTM questionnaire for brushing habits (mean MTM=92.15). Lower scores were observed for those who brushed twice daily four to six or less times in the week (mean MTM=83.18), followed by those who brushed twice daily one to three or less times in the week mean (MTM=78.67), and who never brushed twice daily in the week (mean MTM=75.74). Dunnett’s post hoc analyses revealed significant differences between all groups (p<0.05).

### Hierarchical multiple regression

To examine the relationship between MTM constructs and the initiation and sustenance of twice daily brushing behavior, hierarchical multiple regression models were constructed. Twice daily brushing was taken as the dependent variable and was stepwise regressed. The hierarchical multiple regression revealed that at stage one, behavioral confidence contributed significantly to the regression model, (F (1,227)=33.227, p<0.001) and accounted for 12.4% of the variation in brushing twice daily. Introducing the variable of academic progression explained an additional 15.6% of variation in twice brushing and this change in R ^2^ was significant (F (2,226)=22.054, p<0.001). Finally, the addition of sleeping habits to the regression model explained an additional 17.2 % of the variation and the change in R ^2^ square was also significant (F (3,225)=16.749, p<0.001). Together the three independent variables accounted for 45.2% of the variance in the dependent variable, as shown in Table 4.

**Table 4.** Hierarchical regression analysis of factors effecting twice daily brushing (dependent variable) and independent variables from demographic variables, multi theory model domains, personality inventory and sleeping habits.

Model	R	R Square	Adjusted R Square	Std. Error of the Estimate	Change Statistics		P Value
					R Square Change	F Change	
Behavioral Confidence	0.358	0.128	0.124	0.432	0.128	33.277	<0.001
Behavioral Confidence, Academic	0.404	0.163	0.156	0.424	0.035	9.574	<0.001
Behavioral Confidence, Academic, Sleeping habits	0.427	0.183	0.172	0.420	0.019	5.299	<0.001

## Discussion

The objective of this study was to predict the factors leading to the initiation and sustenance of brushing twice daily behaviour using the constructs of the MTM among college students pursuing health sciences. The theory states that health behaviour change can be measured by two aspects *i.e* initiation and sustenance of the behaviour. MTM theory is applied in many contexts in modifying behaviour in the field of physical activity, dietetics and smoking cessation. With such varied use this is, to our knowledge, the first use of this theory in dentistry. ^11^^,^^12^^,^^18^^,^^19^ The instrument was designed in English instead of the local colloquial dialect keeping in mind the feasibility and practicality of using the MTM. A reliability and validity analysis of the questionnaire was assessed in a pilot study in a similar population. Reliability analysis reported a Cronbach’s alpha value of 0.892. Split-half reliability and Guttman split-half reliability were found to be 0.779 and 0.677, respectively. Test-retest reliability was measured to be 0.779 (p<0.01). ^20^

A total of 45 out of 68 (66%) medical students who answered the questionnaire reported they brushed twice daily less than 3 times a week. In contrast, only 78 out of 161 (48%) dental students brushed twice daily less than 3 times a week. The reason for this could be that dental students were more aware about oral hygiene measures and followed them regularly when compared to their medical counter parts. Similar findings were noted by Cortes *et al*. ^21^ However, Zadik *et al*. in their study to test oral self-care habits of dental and healthcare providers in Israel revealed that dental and medical practitioners had similar frequencies of brushing twice daily which are not in compliance with the finding of the current study. ^10^ However Zadik *et al.* did show overall dental practitioners had a better oral hygiene maintenance practices compared to medical practitioners. ^22^ Kumar *et al*. while testing the dental health behavior in correlation to caries status among medical and dental undergraduate students in India, observed that 56.4% dental students brushed twice daily and only 38.5% medical students brushed twice daily, thus supporting the results of the present study. ^23^

The relation between brushing habits and class year was significant. In total 56% of first years and 64% second year students revealed that they brushed twice daily less than 3 times a week. Whereas less than 50% of clinical students (years 3, 4 and interns) reported that they brushed twice daily less than 3 times a week. As the participant advances in class year, their understanding regarding oral hygiene behavior also improves, suggesting a strong relation between the two variables. Ozalp *et al*. reported that oral health knowledge among dental students in Turkey was significantly higher among fifth year students as compared to fourth years thus supporting the findings of the current study. ^24^

A significant relation between MTM scale and course (Medical or Dental) was also observed. Of the 68 medical students, 24 (35%) of them have received a score of ranging 81-120 on the MTM scale, whereas 116 out of 161 (77%) dental students have a score of 81-120 on the MTM scale. Hence, dental students in the study are more likely to brush twice daily when compared with medical students. A significant relation was observed between academic year and MTM scale as 87% interns scored in the range of 81-120 on the MTM scale. Whereas only 64% first years and 50% of second years revealed similar scores on the MTM scale. Hence fifth year (interns) students have a higher chance of initiating and sustaining the behavior of brushing twice daily when compared to earlier academic years which is similar to the earlier findings.

While using the MTM to predict initiation and sustenance of physical activity among college students, Nahar *et al* reported values to be much lesser in comparison to the present study. ^12^ While assessing the multi theory model to predict initiation and sustenance of a small portion diet among college students, Sharma *et al*. also reported values to be much less in comparison to the present study. ^11^ The reason for this may be that physical activity or maintaining a small portion diet may not be considered as compulsory behaviours and may not be followed by many. On the other hand, brushing is mostly considered as an obligatory activity, thus suggesting the increase in the values of the descriptive analysis. Hence, the MTM fits well to predict the initiation and sustenance of brushing twice daily.

The stepwise Hierarchical regression model suggested that behavioral confidence was the main factor among all demographics, domains of MTM and associated factors, which was followed by academics and sleeping habits **.** The behavioral confidence domain enquired about their level of confidence and conviction of participants to brush twice daily and as explained by Sharma. ^4^ This domain pertains to changing a health behavior so it is not about the immediate present but about perceivable future. Many previous studies based on smoking cessation and diet have demonstrated a strong affiliation to this domain. ^18^ Behavioral confidence seems to form an important foundation in initiation of any behavioral change in a population. The intention to brush and ‘social influences’ and ‘self-efficacy’ are also important indicators for brushing in children and adolescent population and are broadly part of behavioral confidence. ^25^^–^^27^

The academic performance and oral hygiene are also subject of debate as it is observed in some studies that academic performance along many behavioral factors contribute to initiation of brushing in children and adolescents and might be related to their intelligence and application of the same to improve health behavior. ^28^^,^^29^ Sleeping habits are also an important factor in a twice daily brushing routine; studies have proved that brushing at night is an important factor in achieving good quality sleep in children and adolescents. ^30^

### Limitations

As this is an explorative study, random sampling of subjects was not feasible hence does not define the population in general. Since more than two thirds of the study population were dental students, the results of the study might have been affected. But since this is the first time the MTM has been utilized in the field of dentistry, it was appropriate to have a study sample comprising of future health professionals, to confirm the validity of the instrument in the population. In future studies, a much larger sample size should be employed and interventions can be designed for participants who do not follow brushing twice daily behaviour and are willing to initiate and sustain changed brushing behaviour. Since this is a cross-sectional analysis, the responses of the participants are only pertaining to the week before they answered the questionnaire (June 2019).

## Conclusions

The multi theory model is a good tool in predicting the initiation and sustenance of twice daily brushing behaviour among college students pursuing health sciences. All construct of initiation and sustenance significantly influence the prediction of carrying out brushing behaviour and brushing habits of the participants, as students’ progress through their classes in the dental and medical schools they are more likely to indulge in good brushing habits. This may be due to improved knowledge on the subject. This study definitely opens further vistas of research in utilising this novel theory in differing venues in the field of dentistry.

### Ethical statement

Ethical clearance was obtained from the Institutional Ethics Committee of. (Ref No. 17133).

### Informed consent

Informed consent was obtained from all individual participants included in the study. Written informed consent was obtained from the study participants prior to the distribution of questionnaire.

## Data availability

### Underlying data

Figshare: Novel behavioral model in evaluating initiation and sustenance of brushing behavior among students pursuing health sciences. https://doi.org/10.6084/m9.figshare.17209718 ^31^

This project contains the following underlying data:

- Dheeraj with brushing.xlsx (Raw data from questionnaire responses)
  o DATA KEY
  o PD_AD_: Participatory dialogues- advantages
  o PD_DA_: Participatory dialogues- disadvantages
  o BC: Behavioral Confidence
  o PHYSICA_Env: Change in Physical Environment
  o EMOT_Tra: Emotional Transformation
  o Prac_Cha: Practice for Change
  o C_SOC_ENV: Change in Social Environment
  o PI:Personality inventory
  o SH: Sleeping habits

### Extended data

Figshare: Novel behavioral model in evaluating initiation and sustenance of teeth brushing behavior among students pursuing health sciences: a cross-sectional study. https://doi.org/10.6084/m9.figshare.19312103.v1 ^32^

- MTM Questionnaire.docx (Example of questionnaire used).
- MTM DATA extended exel.xlsx (Data used for the validation of the sleep survey). ^20^

Data are available under the terms of the Creative Commons Zero “No rights reserved” data waiver (CC0 1.0 Public domain dedication).
