# Supplementary material for: Novel behavioral model in evaluating initiation and sustenance of teeth brushing behavior among students pursuing health sciences: a cross-sectional study
Source: F1000Res. 2022 Sep 15;11:389. Originally published 2022 Apr 4. [Version 2] doi: 10.12688/f1000research.103077.2 (PMC9490284; doi:10.12688/f1000research.103077.2)
Supplement: Peer review history

# Peer review: Reviewer response for version 2

**DOI:** 10.5256/f1000research.138606.r150536

**Published:** 2022-09-20

**Authors:** Tarakant Bhagat, Abanish Singh

**Affiliations:** 1 Department of Public Health Dentistry, College of Dental Surgery, B.P. Koirala Institute of Health Sciences, Dharan, Nepal; 2 Department Public Health Dentistry, National Medical College, Birgunj, Nepal

No further comments.

Is the work clearly and accurately presented and does it cite the current literature?

Yes

If applicable, is the statistical analysis and its interpretation appropriate?

Yes

Are all the source data underlying the results available to ensure full reproducibility?

Yes

Is the study design appropriate and is the work technically sound?

Yes

Are the conclusions drawn adequately supported by the results?

Yes

Are sufficient details of methods and analysis provided to allow replication by others?

Yes

Reviewer Expertise:

Dental Public Health, Dental Caries, Oral Cancer, Periodontal Disease, Oral Health Related Quality of Life

We confirm that we have read this submission and believe that we have an appropriate level of expertise to confirm that it is of an acceptable scientific standard.

# Peer review: Reviewer response for version 1

**DOI:** 10.5256/f1000research.112730.r129695

**Published:** 2022-06-01

**Authors:** Kumar Gaurav Chhabra

**Affiliations:** 1 Department of Public Health Dentistry, NIMS Dental College and Hospital, NIMS University Rajasthan, Jaipur, India

The study is well designed and methodologically sound. I have some misgivings with the manuscript and hope that the authors will take some encouragement to look at it again

1. What kind of institutions was involved, educational or something else?
2. How was the comparison made by the two experts and refined the single version of the questionnaire?
3. Is the 15 days period sufficient to test the reliability on the same participants? Any supported literature/ document?
4. How was the sample size calculated?
5. The pilot study needs a bit more explanation.
6. Please write with little more details of how this could be implemented in the Indian Scenario. The study is well designed and methodologically sound.

Is the work clearly and accurately presented and does it cite the current literature?

Yes

If applicable, is the statistical analysis and its interpretation appropriate?

Yes

Are all the source data underlying the results available to ensure full reproducibility?

Yes

Is the study design appropriate and is the work technically sound?

Yes

Are the conclusions drawn adequately supported by the results?

Yes

Are sufficient details of methods and analysis provided to allow replication by others?

Yes

Reviewer Expertise:

Various Models of Health Education and how productively they can be implemented.

I confirm that I have read this submission and believe that I have an appropriate level of expertise to confirm that it is of an acceptable scientific standard.

# Response

**Published:** 2022-09-13

**Authors:** Mithun Pai

**Affiliations:** Manipal College Of dental Sciences , Mangalore, India

1. The study was done in a university setting which included a dental and medical students 
2. The experts went through the instrument and suggested changes which were than collated into a single instrument incorporating the changes suggested 
3. The 15 day is sufficient time for test retest analysis and has been explained in reference 20 Dhiraj Panjwani, Mithun BH Pai, Shweta Yellapurkar1, Aayush Anand Poddar, Gururaghavendran Rajesh MS. A Novel Behavioral Model in Initiation and Sustenance of Toothbrushing Behavior among Dental and Medical Students in India: An Exploratory Analysis. J Nat Sc Biol Med. 2021;12:149-54.
4. The sample size calculated was calculated using a G power software and explained in detail in the main article 
5. The pilot study is mentioned in reference 20 
6. The study demonstrated the use of a new model in predicting the initiation and sustenance of twice daily brushing behavior among college students in India hence it might pave way for knowing constraints and barriers for the same and help us in implementing the brushing behavior in adolescents for maintenance of healthy life style 

# Peer review: Reviewer response for version 1

**DOI:** 10.5256/f1000research.112730.r129692

**Published:** 2022-04-19

**Authors:** Tarakant Bhagat, Abanish Singh

**Affiliations:** 1 Department of Public Health Dentistry, College of Dental Surgery, B.P. Koirala Institute of Health Sciences, Dharan, Nepal; 2 Department Public Health Dentistry, National Medical College, Birgunj, Nepal

I appreciate the opportunity to review the article entitled “Novel behavioral model in evaluating initiation and sustenance of teeth brushing behavior among students pursuing health sciences: a cross-sectional study”. This is an interesting study. However, a few minor rectifications may be required as follows:

1. There are few grammatical and punctuation errors in the abstract and introduction sections. Kindly read the manuscript and correct.
2. Introduction is well presented.
3. Methods:
  - The reliability and validity of the questionnaires should be mentioned here (rather than in the discussion section).
  - Please mention clearly about the response rate of the participants on first contact and if the study participants were again contacted for non-response.
  - The pilot study participants were from the same college or different? If from the same college then those participants might have influenced the main study participants. Kindly clarify.
  - The conventional practice is to report p value <0.05 as significant. You have mentioned p=0.05 as significant. Please clarify.
4. Results: The tables should be self-explanatory. Please mention what the “bold letters” in the tables 1 and 2 mean in the footnotes.
5. Discussion: Please mention about the reliability and validity of the tool in methodology section. Not in discussion. 

Is the work clearly and accurately presented and does it cite the current literature?

Yes

If applicable, is the statistical analysis and its interpretation appropriate?

Yes

Are all the source data underlying the results available to ensure full reproducibility?

Yes

Is the study design appropriate and is the work technically sound?

Yes

Are the conclusions drawn adequately supported by the results?

Yes

Are sufficient details of methods and analysis provided to allow replication by others?

Yes

Reviewer Expertise:

Dental Public Health, Dental Caries, Oral Cancer, Periodontal Disease, Oral Health Related Quality of Life

We confirm that we have read this submission and believe that we have an appropriate level of expertise to confirm that it is of an acceptable scientific standard, however we have significant reservations, as outlined above.

# Response

**Published:** 2022-09-13

**Authors:** Mithun Pai

**Affiliations:** Manipal College Of dental Sciences , Mangalore, India

1. The response rate is added as percentage
2. The table footnotes are clarified
3. The query regarding the pilot study and main study participants have been clarified.
4. The reliability and validity of the instrument is added in methods 
5. The required corrections are made.

Thank you
